# Long Distance Dispersal and Connectivity in Amphi-Atlantic Corals at Regional and Basin Scales

**DOI:** 10.1371/journal.pone.0022298

**Published:** 2011-07-22

**Authors:** Flavia L. D. Nunes, Richard D. Norris, Nancy Knowlton

**Affiliations:** 1 Laboratory of Artificial and Natural Evolution, University of Geneva, Geneva, Switzerland; 2 Center for Marine Biodiversity and Conservation, Scripps Institution of Oceanography, University of California San Diego, La Jolla, California, United States of America; 3 National Museum of Natural History, Smithsonian Institution, Washington, D.C., United States of America; Biodiversity Insitute of Ontario - University of Guelph, Canada

## Abstract

Among Atlantic scleractinian corals, species diversity is highest in the Caribbean, but low diversity and high endemism are observed in various peripheral populations in central and eastern Atlantic islands and along the coasts of Brazil and West Africa. The degree of connectivity between these distantly separated populations is of interest because it provides insight into processes at both evolutionary and ecological time scales, such as speciation, recruitment dynamics and the persistence of coral populations. To assess connectivity in broadly distributed coral species of the Atlantic, DNA sequence data from two nuclear markers were obtained for six coral species spanning their distributional ranges. At basin-wide scales, significant differentiation was generally observed among populations in the Caribbean, Brazil and West Africa. Concordance of patterns in connectivity among co-distributed taxa indicates that extrinsic barriers, such as the Amazon freshwater plume or long stretches of open ocean, restrict dispersal of coral larvae from region to region. Within regions, dispersal ability appears to be influenced by aspects of reproduction and life history. Two broadcasting species, *Siderastrea siderea* and *Montastraea cavernosa*, were able to maintain gene flow among populations separated by as much as 1,200 km along the coast of Brazil. In contrast, brooding species, such as *Favia gravida* and *Siderastrea radians*, had more restricted gene flow along the Brazilian coast.

## Introduction

The ability to disperse over long distances and maintain connectivity over large geographical areas has important repercussions for the population dynamics of a species. Over ecological time scales, connectivity contributes to the persistence of populations [1], [2], [3], as the influx of migrants from neighboring healthy ecosystems can buffer mortality due to local disturbances. Large populations are expected to preserve more genetic variety than smaller populations which are susceptible to loss of genetic diversity through the effects of genetic drift [4]. Populations interconnected by regular dispersal and gene flow can behave as large populations and bypass the negative effects of drift. On the other hand, isolated populations can become locally adapted and the origination of new species can occur over evolutionary time scales [5], [6], contributing to the overall diversity of a region. Peripheral populations, those found towards the edges of a species range, are more likely to have reduced numbers of individuals and lower genetic diversity because they are isolated from the central core and because they are found at the environmental limits of the species range [7]. Understanding connectivity with respect to peripheral populations can provide insight into (1) how these populations are able to persist despite withstanding suboptimal conditions and (2) whether they can contribute evolutionary novelty and distinctiveness to the overall population.

Many marine organisms have broad geographical distributions, but it is unclear whether these distributions reflect the ability to maintain frequent long-distance dispersal or if populations are fragmented across their range. In the Atlantic, the most extensive and diverse tropical reef ecosystems are found in the Caribbean Sea, but reefs with low overall species richness yet high endemism are also found along the coast of Brazil, on mid-Atlantic islands and in West Africa [8], [9], [10]. Species whose distribution spans the Atlantic provide an interesting natural experiment to explore basin-scale dispersal in benthic marine organisms and the role of peripheral populations in the evolution of biodiversity.

Dispersal in benthic marine organisms can be achieved in various ways. Long-lived pelagic larvae are capable of traversing hundreds of kilometers [11] either by feeding in the water column or by relying on nutritional resources imparted by the parents. Rafting of recruits or adults on floating debris [12] also allows organisms to colonize distant locations. Habitat preferences also play a role [13], and species able to survive in a wide range of environments or whose habitat is large and continuous may maintain dispersal over long distances despite shorter larval durations, because dispersal can be achieved as a series of smaller steps. Oceanographic currents can either aid or prevent dispersal of larvae. Physical properties of water masses affect larval survival, and dispersal may be limited by temperature, salinity or nutrient content [14]. Dispersal can also be limited by the absence of suitable substrate or chemical cues for settlement [15], [16], [17] because successful recruitment is dependent not only on reaching a destination, but also the ability to settle and become established. Comparative studies across species with different reproductive strategies and life histories can help to clarify which characteristics contribute to successful long-distance dispersal, and whether dispersal is limited by intrinsic characteristics of a single species or extrinsic barriers affecting multiple species.

Corals support biodiversity in the oceans by providing the substrate and architecture for a host of other marine species. Understanding the processes that limit or promote dispersal in coral species can provide insight into how and why populations persist and evolve. Although connectivity and dispersal have been explored for a number of co-distributed Pacific corals [18], [19], basin-scale connectivity among Atlantic corals has only been explored among populations of one scleractinian species [20] and one species of octocoral [21]. However, it is unclear whether the patterns observed in these two species can be generalized to all corals, or whether they are specific to the dispersal ability of these two organisms. Comparative studies across multiple coral species over similar spatial scales can help elucidate this problem.

Corals exhibit a variety of reproductive strategies, making them an interesting group for evaluating the role of reproductive traits in determining dispersal potential. Corals reproduce sexually either by internal fertilization and brooding of larvae or by external fertilization of broadcast gametes followed by development in the water column [22]. Various traits aid long-distance dispersal, such as larval longevity, delayed time to competency, and reproductive output. Prolonged longevity allows larvae to be transported greater distances, and the available nutritional resources will affect the larva's ability to remain alive. Large eggs, such as those observed for many broadcasting species [23], [24], or larvae harboring zooxanthellae, such as for many brooded species [25], may have improved chances of survival. Coral larval longevity can reach upwards of two to three months for some species [26], [27], but many larvae will settle upon encountering suitable substrate much sooner. Brooded larvae are more advanced in their development when released and are competent for settling in 24–48 hours [28], [29], [30]. In contrast, larvae produced from broadcast gametes usually require 5–7 days before being capable of settling [31], [32], although shorter times to settlement competency (54–66 hours) have also been observed in some Pacific species [33]. As time to settlement competency increases, so does the probability of long-distance transport of larvae.

The reproductive output of a coral species may increase its ability for long distance dispersal, simply because the chances of successful dispersal events increase as a greater number of propagules are generated. The number of larvae produced by a colony varies according to colony size, the number of eggs produced per polyp, number of reproductive cycles per year, and fertilization success [32]. The number of reproductive cycles per year may be an important difference between brooding versus broadcasting species, since the former are able to produce larvae over several months of the year to year-round, while the latter typically have only one to three spawning events annually [22], [23], [24]. Finally, sexuality and sex ratio can also play a role in fertilization success. Equal sex ratios, self-compatibility and hermaphrodism may improve chances of fertilization by maximizing the possibility of successful matings. These adaptations can be advantageous in locations were colonies are sparse or where environmental conditions may hinder outcrossing, such as may be expected in peripheral populations.

Species richness and regional endemism vary widely across the Atlantic. Among the 81 species of reef-building corals found in the Atlantic, 68 species are found in the Caribbean, 23 in Brazil and 18 in the Eastern Atlantic (Table S1). There are 12 species endemic to either Brazil or the Eastern Atlantic (five in Brazil, five in West Africa, and two species found in both regions) whereas 49 species are endemic to the Caribbean. Twenty species have wide distributions that span two or more biogeographic regions of the Atlantic, but only nine are found across all three regions: *Madracis asperula*, *M. decactis*, *M. pharensis*, *Montastraea cavernosa*, *Porites astreoides*, *Siderastrea radians*, *S. siderea*, *S. stellata* and the invasive *Tubastraea coccinea*. In addition, *Favia fragum* (Caribbean and West Africa) and *F. gravida* (Brazil and West Africa) were previously considered synonymous [34] and amphi-Atlantic, but recent work indicates these closely related species can be distinguished on the basis of morphology [9] and genetics [35]. It is important to note that species lists and shared occurrences are a work in progress. The shared occurrence of some species across biogeographic regions, such as for *Meandrina braziliensis*, *Siderastrea stellata*, or *Scolymia cubensis*, is debated among taxonomists and biogeographers (for example, [36]). Future work that uses morphological and molecular data from specimens collected in both regions are required to confirm occurrences and refine estimates of endemism for each region.

**Figure 1:**
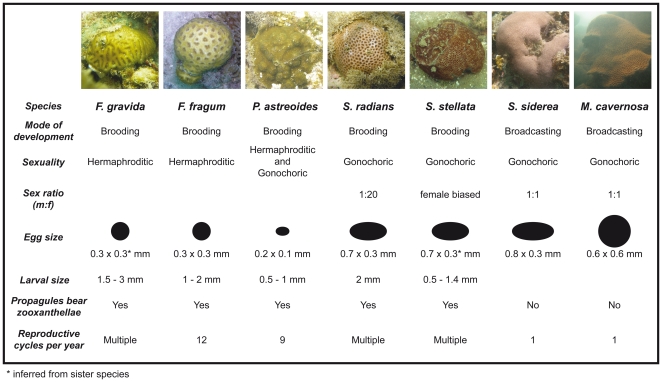
Reproductive traits of studied species: *Favia fragum*, *Favia gravida*, *Porites astreoides*, *Siderastrea radians*, *Siderastrea stellata*, *Siderastrea siderea* and *Montastraea cavernosa*. Summarized from [23], [24], [25], [32], [67], [68], [69], [70]. Images taken by F.N.

Here we provide new genetic data from six coral species together with previously published data from a seventh species; these species have broad geographic distributions across the Atlantic and span a range of reproductive strategies (Fig. 1). The aim of this study was to obtain estimates of basin-wide dispersal and connectivity for a number of Atlantic corals having a variety of biological traits and spanning a wide taxonomic range.

## Materials and Methods

### DNA extraction, amplification and sequencing

Coral tissue samples were collected from colonies of six coral species: *F. fragum*, *F. gravida*, *S. siderea*, *S. radians*, *S. stellata*, and *P. astreoides*. Sampling took place in Panama in 2005, on the island of São Tomé in West Africa in 2006 and in three populations separated by >500 km in Brazil (Abrolhos, João Pessoa and Fortaleza) in 2002 and 2007. Genomic DNA was preserved and extracted following a protocol described previously [35]. Due to the unusually low levels of genetic variation found in the mitochondrial DNA of corals [37], [38], only nuclear loci were used. The intron and exon of *β-tubulin* and the intron of the *Pax-C* gene, which have previously been shown to contain sufficient intraspecific variation in corals for population studies [20], [39], were amplified for all species studied, with the exception of *P. astreoides*, for which only *β-tubulin* could be amplified. For *P. astreoides*, the mitochondrial control region (thought to be one of the most variable regions of the coral mitochondrial genome) was amplified and sequenced for 32 individuals from Panamá and Brazil, but because all individuals contained identical nucleotide sequences at this locus, this marker was not used for further analysis.

Amplification of the two nuclear loci was performed by polymerase chain reaction using published primer sequences for *β-tubulin* [40] and for *Pax-C* [39]. The thermal cycler profile had an initial denaturation step at 94°C for 2 min, followed by 38 cycles of 94°C/45 sec, 50–58°C/45 sec for *β-tubulin* or 50–54°C/45 sec for *Pax-C*, 72°C/90 sec, with a final extension step at 72°C for 5 min. An additional forward primer was designed for the amplification and sequencing of *Pax-C* for *F. fragum* (PaxFsh1: 5′- GGA GGA GCT TGC GAA TAA GA -3′). Multiple bands were amplified in *P. astreoides* and the *Siderastrea* species in *β-tubulin*, and for *S. siderea* in *Pax-C*. Bands at ∼750 bp and ∼600 bp were extracted and purified for products of *β-tubulin* and *Pax-C* respectively, using the Qiaquick Gel Extraction kit (Qiagen). The remaining PCR products were purified either by gel extraction or by elution through a silica-membrane column using the appropriate Qiaquick purification system (Qiagen).

Sequencing of all loci was performed directly on purified PCR products for both forward and reverse directions on an ABI 3130xl genetic analyzer with the BigDye Terminator v3.1 chemistry (Applied Biosystems). Sequence chromatographs were viewed and edited using the Sequencher v4.5 software (Gene Codes Corp). Heterozygous alleles were identified by double peaks observed in sequences from both directions. Indels were observed in the introns of *β-tubulin* for *S. radians* (site 549) and *S. siderea* (site 549), and in sequences of *Pax-C* for *S. radians* (site 134), *F. fragum* (site 205) and *F. gravida* (site 205). Because only a small number of individuals were heterozygous for indels within any species and indel positions were straightforward to identify, indel sites were kept and considered in the analysis.

### Analysis of sequence data

Haplotypes for heterozygous individuals were reconstructed using PHASE v 2.1.1 [41], [42]. The algorithm was run three times for 100 iterations with ten thinning interval steps and 100 burn in steps. The best pairs of haplotypes for each individual resulting from the run with the highest average value for the goodness of fit were used for each nuclear locus.

Haplotype frequencies, molecular diversity indices, tests of neutrality, analysis of molecular variance (AMOVA) and estimates of population differentiation were calculated using Arlequin v.3.11 [43]. Analysis of molecular variance (AMOVA) was used to estimate levels of genetic differentiation among populations of each coral species. One AMOVA was used to estimate differentiation among all populations sampled in the Caribbean, Brazil and West Africa (Φ_ST ALL_). In this AMOVA, populations of *F. fragum* (n = 1) and *F. gravida* (n = 3) were pooled to assess levels of genetic differentiation between these two closely related species. A second AMOVA tested for differentiation among populations within Brazil, the only region for which multiple populations were sampled (Φ_ST BR_). Estimates of pairwise φst used distances between haplotypes. Parsimony haplotype networks were constructed with TCS [44]. Recombination in the two nuclear loci was inferred as reticulations in the haplotype network and tested by the four-gamete test in DnaSP [45]. For clarity, loops were omitted from the haplotype networks and only internal branches are shown. Due to the evidence of recombination, analyses using a coalescent framework (which assume no recombination) were not attempted.

## Results

A total of 238 individuals across all species were sequenced for *β-tubulin* and 191 for *Pax-C*. Haplotype counts for each species can be found in Table S2 and the genotype for each individual is listed in Table S3. Sequences have been deposited in Genbank (see Table S4 for accession numbers).

Interestingly, individuals identified in the field as *S. stellata* in Brazil contained alleles that were either (1) identical or had 1–2 mutations difference to alleles of *S. siderea* from Caribbean or West Africa, or (2) had a combination of haplotypes identified in *S. siderea* and *S. radians* at one or both loci. The presence of *S. siderea* in Brazil has been debated [8], [9], [46], but these results confirm its presence in Brazil, though morphologic differences may exist between regions. Furthermore, the finding that some individuals contain a combination of *S. siderea* and *S. radians* haplotypes indicates that *S. stellata* could be the result of hybridization between these two species. Although most individuals with hybrid genotypes were found in Brazil (n = 20), one hybrid was found in Panamá and four were found in São Tomé. Further investigation is required to confirm the hybrid status of *S. stellata*. For the purposes of this study, data collected from hybrid individuals were kept in the analysis, but only populations of *S. radians* and *S. siderea* were considered. Therefore, haplotypes obtained from *S. stellata* were included as part of either a *S. radians* or *S. siderea* population, depending on similarity/identity with haplotypes from those species.

The number of alleles, unique haplotypes and segregating sites per population are shown in Table 1, as are standard molecular diversity indices and tests of neutrality for each species and each locus. Gene diversity (*h*), which estimates the probability that two randomly sampled haplotypes in a population are different, ranged from 0–0.863 in *Pax-C* and 0.235–0.986 in *β-tubulin*, indicating that while some populations are dominated by a common haplotype, others have a large diversity of haplotypes. Nucleotide diversity (*π*) ranged from 0.0009–0.0090 in *Pax-C* and 0.0003–0.0158 in *β-tubulin*, and the average number of differences between haplotypes of a population (*k*) ranged from 0.324–3.293 in *Pax-C* and 0.235–10.505 in *β-tubulin*. The mean and standard deviation for each molecular diversity index in each population is shown in Table 1. Populations of *S. siderea* in São Tomé (West Africa) had lower average gene diversity than populations in the Caribbean and Brazil. Reduced gene diversity was also observed for *F. gravida* in West Africa in the *Pax-C* locus; however, this trend was not observed in the *β-tubulin* locus. *S. radians* and *P. astreoides* showed overlap in mean values of gene diversity at both loci across Caribbean, Brazilian and West African populations (Table 1). Mean values of nucleotide diversity (*π*) and average number of differences (*k*) overlap among populations for nearly all species in both loci (Table 1), with the exception of populations of *S. radians* in Panamá, which have greater values of *π* and *k* in the *β-tubulin* locus.

**Table 1:** Molecular diversity indices and tests of neutrality for *Pax-C* and *β-tubulin*.

	*N_a_*	*H*	*s*	*h*			*p*			*k*			Tajima's D	Fu's Fs
***Pax-C***														
***F. fragum***				**454 bp**										
CA1 - Panama	32	3	2	0.619	±	0.044	0.0016	±	0.0014	0.724	±	0.555	1.535	0.886
***F. gravida***				**454 bp**										
BR1 - Abrolhos	26	2	1	0.492	±	0.051	0.0011	±	0.0011	0.492	±	0.436	1.437	1.523
BR2 - João Pessoa	46	2	1	0.394	±	0.063	0.0009	±	0.0009	0.394	±	0.375	1.006	1.407
WA1 - São Tome	40	1	0		0			0			0		0	0
***S. radians***				**367 bp**										
CA1 - Panama	21	5	5	0.491	±	0.127	0.0033	±	0.0024	1.210	±	0.803	−0.213	−0.565
BR1 - Abrolhos	14	2	4	0.528	±	0.064	0.0058	±	0.0038	2.110	±	1.250	2.232	4.844
BR2 - João Pessoa	27	5	8	0.735	±	0.059	0.0090	±	0.0053	3.293	±	1.748	1.610	2.968
BR3 - Fortaleza	20	3	5	0.653	±	0.065	0.0058	±	0.0037	2.105	±	1.225	1.511	3.402
WA1 - São Tome	44	5	8	0.571	±	0.052	0.0061	±	0.0038	2.229	±	1.253	0.896	2.316
***S. siderea***				**366 bp**										
CA1 - Panama	27	8	13	0.863	±	0.035	0.0090	±	0.0053	3.288	±	1.745	−0.085	0.156
BR2 - João Pessoa	29	3	4	0.493	±	0.093	0.0028	±	0.0022	1.034	±	0.710	0.041	1.676
BR3 - Fortaleza	20	5	9	0.600	±	0.101	0.0042	±	0.0029	1.542	±	0.963	−1.348	−0.007
WA1 - São Tome	36	2	3	0.108	±	0.068	0.0009	±	0.0010	0.324	±	0.335	−1.237	0.939
***β-tubulin***														
***F. fragum***				**939 bp**										
CA1 - Panama	36	4	5	0.533	±	0.088	0.0014	±	0.0010	1.321	±	0.842	0.248	1.340
***F. gravida***				**939 bp**										
BR1 - Abrolhos	38	2	1	0.235	±	0.081	0.0003	±	0.0003	0.235	±	0.278	−0.020	0.455
BR2 - João Pessoa	52	3	3	0.604	±	0.034	0.0013	±	0.0009	1.190	±	0.775	1.623	2.695
WA1 - São Tome	40	3	2	0.650	±	0.038	0.0008	±	0.0007	0.788	±	0.585	1.278	1.260
***S. radians***				**665 bp**										
CA1 - Panama	20	8	22	0.837	±	0.051	0.0158	±	0.0084	10.505	±	4.999	2.631	3.722
BR1 - Abrolhos	6	4	6	0.867	±	0.129	0.0048	±	0.0033	3.200	±	1.918	1.246	0.352
BR2 - João Pessoa	14	4	6	0.495	±	0.151	0.0017	±	0.0013	1.099	±	0.765	−1.499	−0.214
BR3 - Fortaleza	14	3	2	0.703	±	0.062	0.0013	±	0.0011	0.879	±	0.654	1.080	0.586
WA1 - São Tome	40	5	6	0.677	±	0.042	0.0021	±	0.0014	1.363	±	0.860	−0.090	0.531
***S. siderea***				**665 bp**										
CA1 - Panama	38	32	34	0.986	±	0.011	0.0119	±	0.0063	7.933	±	3.771	−0.130	−21.883
BR1 - Abrolhos	10	7	15	0.933	±	0.062	0.0087	±	0.0051	5.778	±	3.020	0.413	−0.394
BR2 - João Pessoa	38	19	21	0.926	±	0.028	0.0079	±	0.0043	5.266	±	2.602	0.180	−5.479
BR3 - Fortaleza	24	8	16	0.844	±	0.044	0.0062	±	0.0036	4.152	±	2.140	−0.110	0.657
WA1 - São Tome	40	5	5	0.236	±	0.088	0.0005	±	0.0006	0.342	±	0.346	−1.804	−3.315
***P. astreoides***				**596 bp**										
CA1 - Panama	24	4	10	0.728	±	0.045	0.0078	±	0.0044	4.649	±	2.361	2.455	5.884
BR2 - João Pessoa	42	4	12	0.628	±	0.041	0.0085	±	0.0047	5.087	±	2.518	2.515	8.829

*N_a_* is the number of sampled alleles, *H* is the number of unique haplotypes observed, *s* is the number of segregating sites, *h* is the gene diversity, *π* is the average nucleotide diversity and *k* is the average number of nucleotide differences. Biogeographic regions are denoted as CA for Caribbean, BR for Brazil and WA for West Africa. Statistically significant values (α = 0.05) are highlighted in bold.

Tajima's D and Fu's Fs suggest that the loci used are evolving according to neutral expectations for most populations. Two populations (*S. radians* in João Pessoa and *S. siderea* in São Tomé) had significantly negative values of Tajima's D (Table 1), suggesting deviations from neutral expectations. Negative values of Fu's Fs were observed for three populations of *S. siderea* for the *β-tubulin* locus, but only the population of São Tomé also had a significant negative value of Tajima's D (Table 1). Fu's Fs is sensitive to changes in demography, and large negative values in *S. siderea* are indicative of population expansion. Aside from the exceptions mentioned above, it appears that both loci are evolving according to neutral expectations for the remaining populations.

**Figure 2:**
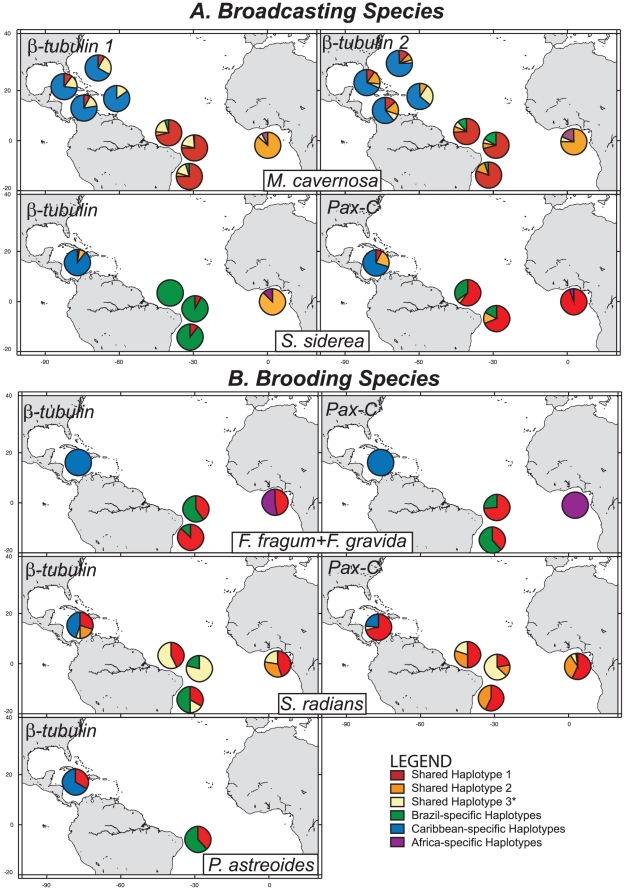
Haplotype frequency pie charts for coral populations across the Atlantic for (A) broadcasters and (B) brooders. The three most commonly occurring haplotypes shared between regions for each species and locus have been coded with the same color (see legend). Private alleles for each geographic region have been binned into one category for clarity. *For *M. cavernosa*, “Shared Haplotype 3” is a sum of the frequencies of all haplotypes shared in two of the three regions.

Significant differentiation among populations was observed for nearly all of the studied species at both loci (Φ_ST ALL_ = 0.142–0.754, p<0.05), except for *P. astreoides* (Φ_ST ALL_ = 0.026, p>0.05). The strongest levels of differentiation were observed among the pooled populations of *F. fragum* and *F. gravida* (Tables 2 and 3), as would be expected for an inter-species comparison. Furthermore, there was significant intra-regional differentiation between populations of the brooders *F. gravida* and *S. radians* of Brazil (Φ_ST BR_ = 0.148–0.582, p<0.05), but not among populations of the broadcasters *S. siderea* or *M. cavernosa* within Brazil (Φ_ST BR_ = −0.026–0.018, p>0.05) (Table 2). Significant population differentiation was observed among nearly all pairwise comparisons (Table 3), with only a few exceptions: (1) populations of *S. siderea* within Brazil were not differentiated at both loci, (2) no significant differentiation was observed between populations of *P. astreoides* in Brazil and the Caribbean and (3) populations of Abrolhos and Fortaleza (both in Brazil) and São Tomé in *S. radians* were not significantly different at *Pax-C*, most likely because similar allele frequencies were observed for two common haplotypes (SRP1 and SRP2). Haplotype frequency pie charts were plotted for each locus and each species on a map to illustrate differences across populations (Fig. 2). Since individuals morphologically identified as *F. fragum* were observed only in Panamá, haplotype frequencies for this population were plotted in the same figure panel as *F. gravida* (absent in the Caribbean) so that a comparison of haplotype frequencies could be made between these two species (Fig. 2).

**Table 2:** Analysis of molecular variance (AMOVA).

A. AMOVA for all populations (Φ_ST ALL_)										
BROODERS										
Species	*Favia fragum+Favia gravia*				*Siderastrea radians*				*Porites astreoides*	
Locus	β-tubulin		Pax-C		β-tubulin		Pax-C		β-tubulin	
	Variance components	% of variation	Variance components	% of variation	Variance components	% of variation	Variance components	% of variation	Variance components	% of variation
Among populations	0.925	67.2	0.576	75.44	0.962	36.77	0.187	14.21	0.067	2.61
Within populations	0.452	32.8	0.187	24.56	1.654	63.23	1.129	85.79	2.465	97.39
Fixation Index Φst	**0.672**		**0.754**		**0.368**		**0.142**		0.026	

BROADCASTERS								
Species	*Siderastrea siderea*				*Montastraea cavernosa*			
Locus	β-tubulin		Pax-C		β-tubulin 1		β-tubulin 2	
	Variance components	% of variation	Variance components	% of variation	Variance components	% of variation	Variance components	% of variation
Among populations	1.090	32.74	0.210	22.59	0.492	14.22	0.682	28.71
Within populations	2.239	67.26	0.718	77.41	2.969	85.78	1.692	71.29
Fixation Index Φst	**0.327**		**0.226**		**0.142**		**0.287**	

B. AMOVA for populations in Brazil (Φ_ST BR_)								
BROODERS								
Species	*Favia gravia*				*Siderastrea radians*			
Locus	β-tubulin		Pax-C		β-tubulin		Pax-C	
	Variance components	% of variation	Variance components	% of variation	Variance components	% of variation	Variance components	% of variation
Among populations	0.362	47.85	0.056	20.8	0.936	58.17	0.228	14.84
Within populations	0.394	52.15	0.215	79.2	0.673	41.83	1.307	85.16
Fixation Index Φst	**0.479**		**0.208**		**0.582**		**0.148**	

BROADCASTERS								
Species	*Siderastrea siderea*				*Montastraea cavernosa*			
Locus	β-tubulin		Pax-C		β-tubulin 1		β-tubulin 2	
	Variance components	% of variation	Variance components	% of variation	Variance components	% of variation	Variance components	% of variation
Among populations	0.045	1.79	−0.015	−2.55	−0.015	−0.86	−0.012	−1.29
Within populations	2.481	98.21	0.620	102.55	1.696	100.86	0.920	101.29
Fixation Index Φst	0.018		−0.026		−0.009		−0.013	

AMOVA was used to estimate levels of genetic differentiation among (A) all populations across all biogeographic regions (Φ_ST ALL_) and (B) among populations within Brazil (Φ_ST BR_), the only region for which multiple populations were sampled. Note that in (A) *F. fragum* and *F. gravida* have been analyzed together to estimate differentiation across these closely related species. Statistically significant values (α = 0.05) are highlighted in bold.

**Table 3:** Pairwise φst for each nuclear locus, for each species.

BROODERS																			
	***F. fragum+F. gravida***	**β-tubulin**									***F. fragum+F. gravida***	**Pax-C**							
		1	2	3	4							1	2	3	4				
1	Abrolhos, Brazil									1	Abrolhos, Brazil								
2	João Pessoa, Brazil	0.479*								2	João Pessoa, Brazil	0.208*							
3	Bocas del Toro, Panamá	0.804*	0.755*							3	Bocas del Toro, Panamá	0.804*	0.809*						
4	Sao Tomé, West Africa	0.218*	0.361*	0.757*						4	Sao Tomé, West Africa	0.660*	0.230*	0.871*					
	***Siderastrea radians***	**β-tubulin**									***Siderastrea radians***	**Pax-C**							
		1	2	3	4	5						1	2	3	4	5			
1	Abrolhos, Brazil									1	Abrolhos, Brazil								
2	João Pessoa, Brazil	0.338*								2	João Pessoa, Brazil	**0.193**							
3	Fortaleza, Brazil	0.463*	0.720*							3	Fortaleza, Brazil	−0.010	0.209*						
4	Bocas del Toro, Panamá	0.17484	0.358*	0.323*						4	Bocas del Toro, Panamá	**0.192**	0.271*	**0.095**					
5	São Tomé, West Africa	0.277*	0.521*	**0.167**	0.393*					5	Sao Tomé, West Africa	−0.040	0.196*	−0.013	**0.125**				
	***Porites astreoides***	**β-tubulin**																	
		1	2																
1	João Pessoa, Brazil	0																	
2	Bocas del Toro, Panamá	0.02611	0																

BROADCASTERS																			
	***Siderastrea siderea***	**β-tubulin**									***Siderastrea siderea***	**Pax-C**							
		1	2	3	4	5						1	2	3	4				
1	Abrolhos, Brazil																		
2	João Pessoa, Brazil	−0.042								1	João Pessoa, Brazil								
3	Fortaleza, Brazil	0.037	0.03906							2	Fortaleza, Brazil	−0.026							
4	Bocas del Toro, Panamá	**0.065**	0.122*	0.160*						3	Bocas del Toro, Panamá	0.253*	0.214*						
5	Sao Tomé, West Africa	0.735*	0.597*	0.748*	0.359*					4	Sao Tomé, West Africa	0.087*	0.097*	0.370*					
	***Montastraea cavernosa***	**β-tubulin 1**									***Montastraea cavernosa***	**β-tubulin2**							
		1	2	3	4	5	6	7	8			1	2	3	4	5	6	7	8
1	Abrolhos, Brazil									1	Abrolhos, Brazil								
2	João Pessoa, Brazil	0.000								2	João Pessoa, Brazil	0.000							
3	Fortaleza, Brazil	−0.017	−0.010							3	Fortaleza, Brazil	−0.020	−0.016						
4	Belize	**0.104**	0.084*	0.109*						4	Belize	0.165*	0.221*	0.189*					
5	Bermuda	**0.083**	**0.060**	**0.089**	−0.010					5	Bermuda	**0.127**	0.218*	0.170*	−0.011				
6	Bocas del Toro, Panamá	**0.113**	0.102*	0.129*	−0.004	−0.005				6	Bocas del Toro, Panamá	**0.129**	0.182*	0.147*	−0.001	−0.007			
7	Puerto Rico	**0.156**	0.137*	0.164*	0.000	0.007	0.008			7	Puerto Rico	0.236*	0.294*	0.257*	0.008	0.021	0.020		
8	São Tomé, West Africa	0.595*	0.458*	0.514*	0.303*	0.423*	0.339*	0.343*		8	São Tomé, West Africa	0.755*	0.780*	0.757*	0.427*	0.514*	0.547*	0.444*	

Statistically significant p-values (α = 0.05) are highlighted in bold, * denotes significant values after Bonferroni correction.

**Figure 3:**
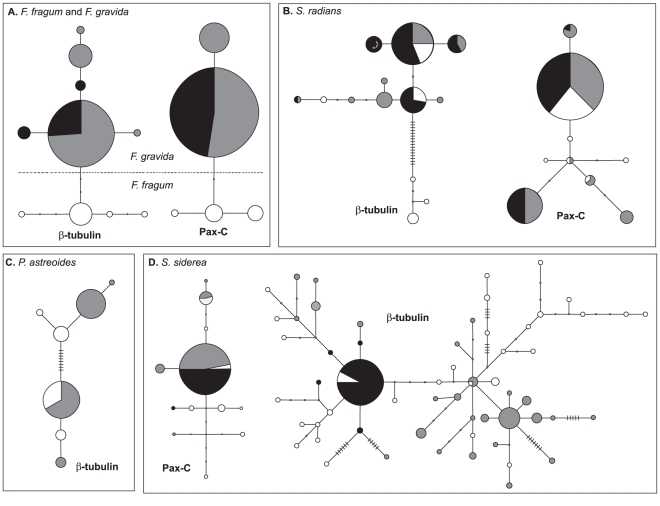
Parsimony haplotype network for (A) *F. fragum* and *F. gravida*, (B) *S. radians*, (C) *P. astreoides* and (D) *S. siderea*. Haplotypes observed in the Caribbean, Brazil and West Africa are shown as white, grey and black circles, respectively. The size of each circle reflects the frequency that a haplotype is observed. Notches symbolize intermediate haplotypes not observed.

Statistical parsimony haplotype networks for each species and locus are shown in Fig. 3. Networks for *F. fragum* and *F. gravida* were plotted together to show levels of divergence among haplotypes of the two species. Haplotype networks were typically composed of a small number of haplotypes (H<15), although the network for *β-tubulin* in *S. siderea* had 60 haplotypes. No loops were observed in the haplotype networks of *F. fragum* and *F gravida* for both loci, or in the network of *P. astreoides* for *β-tubulin*. One loop was observed in the networks of *S. radians* at each locus. For *S. siderea*, no loops were observed in *Pax-C*; however, numerous loops were observed in *β-tubulin*, suggesting that in this locus, several recombination events have occurred. The four gamete test indicates that only a few recombination events have occurred at each locus for most species (Rm = 0–4), except for *β-tubulin* of *S. siderea*, where a minimum of 12 recombination events are inferred. Recombination has also been observed in *β-tubulin1* and *β-tubulin2* of *M. cavernosa* [20]. Every haplotype network had 1–5 haplotypes that were shared between two or more regions. Differences between haplotypes from different regions were typically small, on the order of one to a few mutations. There were a relatively high proportion of private alleles (those restricted to a single population) for each locus and species, ranging from 44–87% of the observed haplotypes for a population. The proportion of alleles that were observed only once was low at each locus for most species (0–27%), except for *β-tubulin* in *S.siderea*, where 72% of haplotypes were observed only once.

## Discussion

### Genetic differentiation across regions of the Atlantic

The data collected for six coral species plus an existing dataset for *Montastraea cavernosa* [20] suggest that significant differentiation is present between the Caribbean, Brazil and West Africa for most sampled coral species. Concordance in the pattern of regional isolation occurs at the scale of genes and species. Both nuclear markers show consistent patterns in differentiation, and regional differentiation across multiple species indicates that barriers to gene flow between biogeographic regions in the Atlantic are effective for most corals.

The most likely biogeographic barrier separating Caribbean and Brazilian coral populations are the deltas and low salinity waters of the Amazon, Orinoco and numerous rivers along the coast of northern South America. The Amazon is the largest among them, and accounts for 16% of the annual freshwater discharge into the world's oceans [47]. Its plume extends 200–500 km in width [48] and is recognizable 24–32 m below the surface [49]. The Amazon and Orinoco Rivers attained their current drainage configuration around the Late Miocene [50], indicating that low salinity and high sedimentation may have been a feature of this coastal region for ∼10 Ma. Approximately 2,300 km of coastline rich in soft sediment bottoms and low salinity coastal waters between Caribbean and Brazilian reefs may pose substantial barriers to dispersal, because the substrate near river deltas is inadequate for coral settlement and coral larvae are very sensitive to the changes in salinity [51] that these large river plumes create.

Levels of genetic differentiation between between Caribbean *F. fragum* and South Atlantic *F. gravida* were the most pronounced among all of the studied species (Table 2A and 3). This finding, combined with the observation that the species do not co-occur [9], supports the hypothesis that *F. fragum* and *F. gravida* are distinct [35]. Only a small number of mutations separate *F. fragum* and *F. gravida* (Fig. 3), but this may be due to the recent divergence of the two species coupled with the slow rates of mutation observed in corals [38].

The only species for which no significant differentiation was observed between Caribbean and Brazilian populations was *P. astreoides*. For *β-tubulin* (the only marker amplified for this species, see Methods), *P. astreoides* has one common haplotype that is observed both in Brazil and Panamá at similar frequencies, with all other haplotypes being unique to each respective population (see Fig. 3). This common haplotype may be an ancestral haplotype that has been maintained over time in the two populations without continued gene flow. Sequences of *β-tubulin* for *P. astreoides* have lower variation compared to the other species, perhaps insufficient to detect differences among populations. Additional support from faster-evolving markers such as microsatellites is required to determine whether continued gene flow is maintained between the Caribbean and Brazil in this species. Alternatively, *P. astreoides*, a brooder, may be able to raft or be more tolerant to freshwater and high sedimentation.

Significant regional differentiation was also observed with respect to West Africa (Table 3), suggesting that long distances of open ocean may be impassable for coral larvae. The easternmost point of Brazil and the island of São Tomé are separated by 4,800 km. Some mid-Atlantic islands could serve as stepping-stones for dispersal, decreasing dispersal distances by about one half. However, it appears that even these distances are too great to maintain gene flow or that the populations on these islands (such as the St. Peter and Paul rocks) are too small or ephemeral to be significant in trans-Atlantic dispersal. Interestingly, the population of *M. cavernosa* on the island of Bermuda is able to maintain connectivity with Caribbean populations despite being separated by at least 1,000 km [20]. Presumably the fast-moving currents of the Gulf Stream are able to maintain the influx of larvae to Bermuda. The combined effects of distance and physical oceanography are likely important isolating factors for West African coral populations.

Concordance in patterns of regional connectivity in the Atlantic is not observed in most other species of marine organisms. Four species of sea urchins sharing similar life histories and pelagic larval durations show markedly different patterns of differentiation across the Atlantic. The Amazon appears to be a significant barrier to gene flow for most species [52], [53], [54], but not all [55]. Multiple diagnostic mutations separate eastern and western Atlantic populations of some urchin species [53], [54], while others maintain continued exchange across the South Atlantic [52], [55]. The discordant patterns observed among sea urchins with similar traits in reproduction and dispersal indicate that barriers to gene flow in the Atlantic may be permeable depending on aspects other than reproduction and pelagic larval duration.

Among reef fish, similar discordance has been observed across species. Some species achieve high gene flow throughout the Atlantic (*Myripristis jacobus* [56]), or throughout their range (*Halichoeres garnoti* [57]). Others are highly differentiated [58], [59], while some display intermediate levels of gene flow between these two extremes. Interestingly, broad adult habitat preferences among surgeonfish of the genus *Acanthurus* correlates well with dispersal ability across the Amazon, suggesting that among reef fish, both ecology and reproduction play important roles in dispersal potential [13].

In sum, the similarities in patterns of connectivity observed among various coral species indicate that barriers such as the Amazon or stretches of open ocean are likely impassible for most coral larvae, leading to concordance of patterns. These barriers may be more permeable, however, for other organisms whose ecology and life history permit dispersal at greater distances than corals, resulting in more variable patterns of connectivity observed in other amphi-Atlantic marine organisms.

### Genetic differentiation within regions

Although patterns in inter-regional differentiation are concordant among most coral species, different estimates of gene flow are observed within regions across coral species, and the extent of gene flow is correlated with reproductive traits. In this study, multiple populations within a region were sampled only in Brazil. For each species, two or three populations each separated by >500 km across a total of 2,000 km along the coast of Brazil were sampled for all species except *F. fragum* (present only in the Caribbean) and *P. astreoides*, for which specimens were only taken in João Pessoa (see Fig. 2). Strong differentiation observed at both loci between populations of the brooder *F. gravida* in Abrolhos and João Pessoa (Table 3) indicates that little exchange is occurring between populations of this species along the coast of Brazil. Intermediate levels of differentiation were observed for the brooder *S. radians*, where AMOVA indicates that differentiation is significant between populations within regions for both loci (Table 2), but not all pairwise population comparisons showed significant differentiation (Table 3). In contrast, gene flow appears to be maintained between three populations spanning 2,000 km of Brazilian coast of the two broadcasting corals studied, *S. siderea* and *M. cavernosa* (Table 2).

The two broadcasting species that are able to maintain gene flow along the coast of Brazil are both gonochoric with one reproductive cycle per year, while the two brooding species with fragmented populations are hermaphroditic and spawn multiple times per year (Fig. 1). Egg sizes for broadcasting *M. cavernosa* and *S. siderea* are larger than for the brooder *F. gravida*, but only marginally larger than brooding *S. radians*. However, the dispersing propagules for brooders, the planula larvae, are larger than broadcast eggs and bear zooxanthellae (Fig. 1). The findings of this study indicate that neither the frequency of spawning nor the size of the dispersing propagules (and associated energy reserves) appear to provide an advantage for long distance dispersal. Brooded larvae may allocate much of their energy reserves towards settlement and growth rather than dispersal. Because broadcast gametes must spend time in the water column before being competent for settlement, their chances of being entrained in currents that take them away from their natal reef are greater, resulting in greater dispersal distances.

The species with the greatest population differentiation, *F. gravida*, is a hermaphroditic brooder, possibly capable of self-fertilization like its sibling species *F. fragum* [60]. Self-fertilization may ensure reproductive success when population densities are low and sperm limitation reduces chances of fertilization, but with the disadvantage that populations may become inbred and dominated by clonemates. Self-fertilization will also result in apparent reduced gene flow, so at least part of the high differentiation among populations of *F. gravida* could reflect inbreeding.

Some Caribbean coral species are able to maintain gene flow throughout the Caribbean and Bermuda, such as *M. cavernosa* [20] and *M. faveolata* [61], but dispersal within the Caribbean is more restricted for other species. Among the seven Caribbean corals studied to date, gene flow is restricted to <500 km for most species [62]. *Acropora palmata* [63] and *A. cervicornis* [62] are able to disperse widely, but their populations are subdivided between the eastern and western Caribbean. Gene flow appears to be restricted to even shorter distances for species such as *Agaricia agaricites* [64] and some members of the *M. annularis* species complex [61], [65]. All of the aforementioned Caribbean corals are broadcasters with the exception of *A. agaricites*. In agreement with findings for Brazilian brooders, *A. agaricites* in the Caribbean has a more fragmented population than broadcasting species, although studies on additional Caribbean brooders are required to confirm the generality of this trend.

Among Caribbean broadcasters, there are no clear explanations for differences in dispersal ability. Dispersal ability in *A. palmata* and *A. cervicornis* appears to be more restricted than for *M. cavernosa* and *M. faveolata*, even though Caribbean *Acropora* eggs are at least 1.4 times greater in size [23]. Egg size may not be a good predictor of dispersal ability, as nutritional reserves may be allocated for settlement rather than survival in the water column. Most striking is the difference in dispersal ability among closely related species with similar reproductive traits, such as short-ranging *M. annularis* and Caribbean-wide *M. faveolata* [61].

In the Indo-Pacific, trends in dispersal ability show even less association with reproductive mode. A survey of nine co-distributed species along 1200 km of the Great Barrier Reef showed no correlation between connectivity and reproductive mode [18]. Likewise, reproductive mode was not a good predictor of dispersal ability among several coral populations along the GBR and the peripheral populations on Lord Howe Island [19]. Paradoxically, species with highly different larval longevity estimates and life history characteristics have been observed to have similar levels of fine to meso-scale population subdivision [66].

Determining which life history traits impart greater success in long-distance dispersal is not straightforward. In Atlantic corals, reproductive mode appears to provide an advantage for long-distance dispersal, but this trend is not observed for corals in the Indo-Pacific. These results indicate that single aspects of coral life history, such as reproductive mode, propagule size or larval longevity, cannot alone predict dispersal ability, and that complex interactions of multiple factors likely play a role in determining connectivity. In other organisms, aspects of reproduction and life history, when taken alone, have at times also not been good predictors of dispersal ability and connectivity. In reef fish of the Atlantic, for example, pelagic larval duration is not a good predictor of population connectivity [56].

While dispersal ability is an important factor for maintaining connectivity, environmental factors also play an important role, as migrants must also be able to adapt to the environment at a distant reef in order to become established and contribute to gene flow. Interactions with other organisms, such as competition for space, can also affect successful recruitment. In sum, patterns in connectivity are difficult to predict based on reproductive traits alone and are likely influenced by multiple biotic and abiotic factors.

### Patterns of genetic diversity

Mean values of molecular diversity indices overlap among regions for most coral species, although for some comparisons, diversity appears to be lower in the peripheral populations, such as gene diversity in *S. siderea* of West Africa for both loci and *F. gravida* in West Africa for Pax-C (Table 1). On average, however, there does not appear to be a strong trend in decreasing genetic diversity towards the edges of the species range for most species.

This finding is at odds with patterns observed in populations of the coral *M. cavernosa*, where genetic diversity was found to be lower in Brazil and West Africa compared to the Caribbean [20]. Similarly, reduced allozyme allelic diversity and heterozygosity was also found for several coral species on the isolated Lord Howe Island relative to populations on the Great Barrier Reef [19] as would be predicted by small population size and founder effects. Peripheral populations that are isolated from the center of species range or center of diversity may have smaller effective population size (*N_e_*) and can suffer loss of diversity via genetic drift. In addition, peripheral populations colonized by a small group of founders may only contain a subset of the full diversity of alleles observed in the main population. However, loss of genetic diversity in Brazil and West Africa does not appear to be a consistent trend among the coral species studied here, or if differences in genetic diversity exist among regions, they have not been captured with the markers used here.

Overall, levels of polymorphism and the number of haplotypes for the brooding species *F. fragum*, *F. gravida*, *P. astreoides* and *S. radians* were much lower than for the broadcasters *S. siderea* and *M. cavernosa*. For example, >40 haplotypes were observed for *M. cavernosa* and *S. radians*, while the number of haplotypes observed for the four brooding species ranged from 6–12. High levels of polymorphism in *M. cavernosa* may have made the patterns of reduced variation in the peripheral populations more pronounced and readily detected. In *S. siderea*, decreasing trends in gene diversity (*h*) are observed, but there is overlap in the mean values of other diversity indices among the three biogeographic regions (Table 1).

Regional differences in genetic diversity may have gone undetected, but another reasonable alternative is that loss of variation has occurred in peripheral populations of *M. cavernosa*, but not in other species. Although regional isolation is observed for nearly all species, the mechanisms that lead to reduced variation may be less effective in some species relative to others. Loss of variation resulting from genetic drift is more pronounced in populations with small effective size (*N_e_*). Differences in *N_e_* between the species may result in different rates in the loss of genetic diversity. Founder effects may also be less pronounced if peripheral populations have received multiple founding events. Some of the brooding species studied have multiple mating cycles per year and reproductive strategies that may allow them to establish larger populations over shorter time periods, thereby increasing *N_e_* and buffering the loss of genetic diversity. Another possible scenario is that historically some of the other species were able to maintain gene flow up until more recently, while gene flow for *M. cavernosa* ended a longer time in the past – subjecting the species to a longer period of time for drift to act upon.

### Conclusions

Patterns of connectivity across broad regions of the Atlantic are congruent across multiple species of corals, suggesting that barriers to dispersal such as the Amazon freshwater plume and the long distances that separate the east and west South Atlantic are effective for most coral species. At shorter, regional scales, it appears that some aspects of reproduction such as mode of larval development can influence gene flow, although this pattern cannot be generalized to all scenarios. Broadcasters have greater dispersal ability in the Atlantic, but several exceptions exist in the Indo-Pacific, suggesting that complex interactions between biotic and abiotic factors can limit connectivity rather than single traits of a species. Loss of genetic diversity in peripheral populations of corals is also not a general trend observed among all amphi-Atlantic corals, likely due to differences in population size and timing of isolation across species. Regional isolation may mean that the persistence of peripheral populations relies primarily on local to regional recruitment since migration from other regions must occur rarely. These populations may be more vulnerable to disturbance on ecological time scales as a result of their isolation. On the other hand, isolated peripheral populations with small effective population size may become the sites for local adaptation and allopatric speciation. In both scenarios, these regions require special attention for conservation as a result of their potential vulnerability to environmental change, but also their importance in generating diversity.

## Supporting Information

Table S1 Coral species list for the Atlantic. Species occurrence in each biogeographic region is indicated by an X. Total species count for each region is found at the bottom of each column.
(PDF)

Table S2 Haplotype count for each species, locus and population.
(PDF)

Table S3 Two-locus genotypes for sampled individuals.
(PDF)

Table S4 Genbank accession number for all sequenced haplotypes.
(PDF)
