# Supplementary material for: Long Distance Dispersal and Connectivity in Amphi-Atlantic Corals at Regional and Basin Scales
Source: PLoS One. 2011 Jul 22;6(7):e22298. doi: 10.1371/journal.pone.0022298 (PMC3142122; doi:10.1371/journal.pone.0022298)
Supplement: Table S1 — Coral species list for the Atlantic. Species occurrence in each biogeographic region is indicated by an X. Total species count for each region is found at the bottom of each column. (PDF) [file pone.0022298.s001.pdf]

Species		Caribbean	Brazil	W. Africa
Acropora	*cervicornis*	X		
Acropora	*palmata*	X		
Agaricia	*fragilis*	X	X	
Agaricia	*undata*	X		
Agaricia	*agaricites*	X	X	
Agaricia	*grahamae*	X		
Agaricia	*humilis*	X		
Agaricia	*tenuifolia*	X		
Agaricia	*lamarcki*	X		
Cladocora	*arbuscula*	X		
Cladocora	*debilis*	X		
Colpophyllia	*natans*	X		
Dendrogyra	*cylindrus*	X		
Dichocoenia	*stellaris*	X		
Dichocoenia	*stokesii*	X		
Diploria	*clivosa*	X		
Diploria	*labyrinthiformis*	X		
Diploria	*strigosa*	X		
Eusmilia	*fastigiata*	X		
Favia	*leptophylla*		X	
Favia	*fragum*	X		X
Favia	*gravida*		X	X
Helioseris	*cucullata*	X		
Isophyllastrea	*rigida*	X		
Isophyllia	*sinuosa*	X		
Leptoseris	*cailleti*	X		
Madracis	*asperula*	X	X	X
Madracis	*brueggemanni*	X	X	
Madracis	*carmabi*	X		
Madracis	*auretenra*	X		
Madracis	*decactis*	X	X	X
Madracis	*formosa*	X		
Madracis	*myriaster*	X		
Madracis	*pharensis*	X	X	X
Madracis	*profunda*			X
Madracis	*senaria*	X		
Manicina	*areolata*	X		
Meandrina	*braziliensis*		X	
Meandrina	*danae*	X		
Meandrina	*meandrites*	X		
Montastraea	*annularis*	X		
Montastraea	*faveolata*	X		
Montastraea	*cavernosa*	X	X	X
Montastraea	*franksi*	X		
Mussa	*angulosa*	X		
Mussismilia	*braziliensis*		X	
Mussismilia	*harttii*		X	
Mussismilia	*hispida*		X	
Mycetophyllia	*aliciae*	X		
Mycetophyllia	*danaana*	X		
Mycetophyllia	*ferox*	X		
Mycetophyllia	*lamarckiana*	X		
Mycetophyllia	*reesi*	X		
Oculina	*arbuscula*	X		
Oculina	*diffusa*	X		
Oculina	*patagonica*	X		
Oculina	*robusta*	X		
Oculina	*tenella*	X		
Oculina	*valenciennesi*	X		

Species		Caribbean	Brazil	W. Africa
Oculina	*varicosa*	X		
Porites	*astreoides*	X	X	X
Porites	*bernardi*			X
Porites	*branneri*	X	X	
Porites	*colonensis*	X		
Porites	*divaricata*	X		
Porites	*furcata*	X		
Porites	*hentscheli*			X
Porites	*porites*	X		X
Schizoculina	*africana*			X
Schizoculina	*fissipara*			X
Scolymia	*cubensis*	X	X	
Scolymia	*lacera*	X		
Scolymia	*wellsi*	X	X	
Siderastrea	*stellata*	X	X	X
Siderastrea	*radians*	X	X	X
Siderastrea	*siderea*	X	X	X
Solenastrea	*bournoni*	X		
Solenastrea	*hyades*	X		
Stephanocoenia	*intersepta*	X	X	
Tubastraea	*coccinea*	X	X	X
Tubastraea	*tagusensis*		X	X
TOTAL	81	69	23	18
