# Supplementary material for: Long Distance Dispersal and Connectivity in Amphi-Atlantic Corals at Regional and Basin Scales
Source: PLoS One. 2011 Jul 22;6(7):e22298. doi: 10.1371/journal.pone.0022298 (PMC3142122; doi:10.1371/journal.pone.0022298)
Supplement: Table S2 — Haplotype count for each species, locus and population. (PDF) [file pone.0022298.s002.pdf]

**Table S2.** Haplotype count for each species, locus and population

### *Favia gravida* and *Favia fragum, Pax-C*

| Haplotype | BR1 Abrolhos | BR2 João Pessoa | | CA1 Panamá | WA1 São Tomé |
|---|---|---|---|---|---|
| FFP1 | 16 | 12 | | 0 | 0 |
| FFP2 | 10 | 34 | | 0 | 40 |
| FFP3 | 0 | 0 | | 4 | 0 |
| FFP4 | 0 | 0 | | 15 | 0 |
| FFP5 | 0 | 0 | | 13 | 0 |
| TOTAL | 26 | 46 | | 32 | 40 |

### *Favia gravida* and *Favia fragum, β-tubulin*

| Haplotype | BR1 Abrolhos | BR2 João Pessoa | | CA1 Panamá | WA1 São Tomé |
|---|---|---|---|---|---|
| FFB1 | 33 | 21 | | 0 | 19 |
| FFB2 | 5 | 0 | | 0 | 0 |
| FFB3 | 0 | 6 | | 0 | 0 |
| FFB4 | 0 | 25 | | 0 | 0 |
| FFB5 | 0 | 0 | | 24 | 0 |
| FFB6 | 0 | 0 | | 4 | 0 |
| FFB7 | 0 | 0 | | 4 | 0 |
| FFB8 | 0 | 0 | | 4 | 0 |
| FFB9 | 0 | 0 | | 0 | 9 |
| FFB10 | 0 | 0 | | 0 | 12 |
| TOTAL | 38 | 52 | | 36 | 40 |

### *Porites astreoides - β-tubulin*

| Haplotype | BR2 João Pessoa | CA1 Panamá |
|---|---|---|
| PAB1 | 20 | 0 |
| PAB2 | 16 | 8 |
| PAB3 | 5 | 0 |
| PAB4 | 1 | 0 |
| PAB5 | 0 | 9 |
| PAB6 | 0 | 5 |
| PAB7 | 0 | 2 |
| TOTAL | 42 | 24 |

### *Siderastrea radians, Pax-C*

| Haplotype | BR1 Abrolhos | BR2 João Pessoa | BR3 Fortaleza | CA1 Panamá | WA1 São Tomé |
|---|---|---|---|---|---|
| SRP1 | 8 | 6 | 10 | 15 | 25 |
| SRP2 | 6 | 4 | 6 | 0 | 15 |
| SRP3 | 0 | 12 | 0 | 0 | 1 |
| SRP4 | 0 | 4 | 4 | 0 | 2 |
| SRP5 | 0 | 1 | 0 | 1 | 0 |
| SRP6 | 0 | 0 | 0 | 2 | 0 |
| SRP7 | 0 | 0 | 0 | 2 | 0 |
| SRP8 | 0 | 0 | 0 | 1 | 0 |
| SRP9 | 0 | 0 | 0 | 0 | 1 |
| TOTAL | 14 | 27 | 20 | 21 | 44 |

### *Siderastrea radians, β-tubulin*

| Haplotype | BR1 Abrolhos | BR2 João Pessoa | BR3 Fortaleza | CA1 Panamá | WA1 São Tomé |
|---|---|---|---|---|---|
| SRB1 | 2 | 0 | 0 | 0 | 0 |
| SRB2 | 2 | 0 | 6 | 6 | 18 |
| SRB3 | 1 | 0 | 0 | 0 | 1 |
| SRB4 | 1 | 1 | 0 | 0 | 0 |
| SRB5 | 0 | 10 | 0 | 0 | 0 |
| SRB6 | 0 | 1 | 4 | 0 | 7 |
| SRB7 | 0 | 2 | 0 | 0 | 0 |
| SRB8 | 0 | 0 | 4 | 1 | 1 |
| SRB9 | 0 | 0 | 0 | 5 | 0 |
| SRB10 | 0 | 0 | 0 | 4 | 13 |
| SRB11 | 0 | 0 | 0 | 1 | 0 |
| SRB12 | 0 | 0 | 0 | 1 | 0 |
| SRB13 | 0 | 0 | 0 | 1 | 0 |
| SRB14 | 0 | 0 | 0 | 1 | 0 |
| TOTAL | 6 | 14 | 14 | 20 | 40 |

### *Siderastrea siderea, Pax-C*

| Haplotype | BR2 João Pessoa | BR3 Fortaleza | CA1 Panamá | WA1 São Tomé |
|---|---|---|---|---|
| SSP1 | 20 | 12 | 2 | 34 |
| SSP2 | 5 | 5 | 0 | 0 |
| SSP3 | 4 | 1 | 6 | 0 |
| SSP4 | 0 | 1 | 0 | 0 |
| SSP5 | 0 | 1 | 0 | 0 |
| SSP6 | 0 | 0 | 3 | 0 |
| SSP7 | 0 | 0 | 4 | 0 |
| SSP8 | 0 | 0 | 7 | 0 |
| SSP9 | 0 | 0 | 2 | 0 |
| SSP10 | 0 | 0 | 1 | 0 |
| SSP11 | 0 | 0 | 2 | 0 |
| SSP12 | 0 | 0 | 0 | 2 |
| TOTAL | 29 | 20 | 27 | 36 |

### *Siderastrea siderea, β-tubulin*

| Haplotype | BR1 Abrolhos | BR2 João Pessoa | BR3 Fortaleza | CA1 Panamá | WA1 São Tomé |
|---|---|---|---|---|---|
| SSB1 | 2 | 1 | 1 | 0 | 0 |
| SSB2 | 2 | 1 | 3 | 0 | 0 |
| SSB3 | 1 | 3 | 0 | 1 | 0 |
| SSB4 | 1 | 2 | 0 | 0 | 0 |
| SSB5 | 2 | 3 | 0 | 0 | 0 |
| SSB6 | 1 | 0 | 0 | 0 | 0 |
| SSB7 | 1 | 0 | 0 | 0 | 0 |
| SSB8 | 0 | 9 | 7 | 0 | 0 |
| SSB9 | 0 | 2 | 0 | 0 | 0 |
| SSB10 | 0 | 1 | 0 | 0 | 0 |
| SSB11 | 0 | 2 | 0 | 0 | 0 |
| SSB12 | 0 | 1 | 0 | 0 | 0 |

***Siderastrea siderea, β-tubulin (cont.)***

| Haplotype | BR1<br>Abrolhos | BR2<br>João Pessoa | BR3<br>Fortaleza | CA1<br>Panamá | WA1<br>São Tomé |
|---|---|---|---|---|---|
| SSB13 | 0 | 1 | 0 | 0 | 0 |
| SSB14 | 0 | 1 | 0 | 0 | 0 |
| SSB15 | 0 | 1 | 0 | 0 | 0 |
| SSB16 | 0 | 1 | 0 | 0 | 0 |
| SSB17 | 0 | 1 | 0 | 0 | 0 |
| SSB18 | 0 | 1 | 0 | 0 | 0 |
| SSB19 | 0 | 4 | 1 | 0 | 0 |
| SSB20 | 0 | 1 | 0 | 0 | 0 |
| SSB21 | 0 | 2 | 0 | 0 | 0 |
| SSB22 | 0 | 0 | 3 | 0 | 0 |
| SSB23 | 0 | 0 | 2 | 0 | 0 |
| SSB24 | 0 | 0 | 1 | 0 | 0 |
| SSB25 | 0 | 0 | 6 | 0 | 0 |
| SSB26 | 0 | 0 | 0 | 1 | 0 |
| SSB27 | 0 | 0 | 0 | 1 | 0 |
| SSB28 | 0 | 0 | 0 | 1 | 0 |
| SSB29 | 0 | 0 | 0 | 1 | 0 |
| SSB30 | 0 | 0 | 0 | 1 | 0 |
| SSB31 | 0 | 0 | 0 | 1 | 0 |
| SSB32 | 0 | 0 | 0 | 1 | 0 |
| SSB33 | 0 | 0 | 0 | 1 | 0 |
| SSB34 | 0 | 0 | 0 | 1 | 0 |
| SSB35 | 0 | 0 | 0 | 1 | 0 |
| SSB36 | 0 | 0 | 0 | 3 | 35 |
| SSB37 | 0 | 0 | 0 | 2 | 0 |
| SSB38 | 0 | 0 | 0 | 1 | 0 |
| SSB39 | 0 | 0 | 0 | 1 | 0 |
| SSB40 | 0 | 0 | 0 | 1 | 0 |
| SSB41 | 0 | 0 | 0 | 1 | 0 |
| SSB42 | 0 | 0 | 0 | 1 | 0 |
| SSB43 | 0 | 0 | 0 | 1 | 0 |
| SSB44 | 0 | 0 | 0 | 1 | 0 |
| SSB45 | 0 | 0 | 0 | 1 | 0 |
| SSB46 | 0 | 0 | 0 | 1 | 0 |
| SSB47 | 0 | 0 | 0 | 1 | 0 |
| SSB48 | 0 | 0 | 0 | 1 | 0 |
| SSB49 | 0 | 0 | 0 | 1 | 0 |
| SSB50 | 0 | 0 | 0 | 4 | 0 |
| SSB51 | 0 | 0 | 0 | 1 | 0 |
| SSB52 | 0 | 0 | 0 | 1 | 0 |
| SSB53 | 0 | 0 | 0 | 1 | 0 |
| SSB54 | 0 | 0 | 0 | 1 | 0 |
| SSB55 | 0 | 0 | 0 | 1 | 0 |
| SSB56 | 0 | 0 | 0 | 1 | 0 |
| SSB57 | 0 | 0 | 0 | 0 | 1 |
| SSB58 | 0 | 0 | 0 | 0 | 2 |
| SSB59 | 0 | 0 | 0 | 0 | 1 |
| SSB60 | 0 | 0 | 0 | 0 | 1 |
| TOTAL | 10 | 38 | 24 | 38 | 40 |
