# Supplementary material for: Long Distance Dispersal and Connectivity in Amphi-Atlantic Corals at Regional and Basin Scales
Source: PLoS One. 2011 Jul 22;6(7):e22298. doi: 10.1371/journal.pone.0022298 (PMC3142122; doi:10.1371/journal.pone.0022298)
Supplement: Table S3 — Two-locus genotypes for sampled individuals. (PDF) [file pone.0022298.s003.pdf]

Sample	β-tubulin		Pax-C	
Favia fragum				
Bocas del Toro, Panamá				
P016	FFB 06	FFB 06		
P039	FFB 08	FFB 08	FFP 03	FFP 03
P040	FFB 08	FFB 08	FFP 03	FFP 03
P041	FFB 07	FFB 07	FFP 04	FFP 04
P106	FFB 05	FFB 05	FFP 05	FFP 05
P107	FFB 05	FFB 05	FFP 05	FFP 04
P108	FFB 05	FFB 05	FFP 04	FFP 04
P109	FFB 05	FFB 05	FFP 05	FFP 05
P110	FFB 05	FFB 05	FFP 05	FFP 05
P111	FFB 05	FFB 05		
P112	FFB 07	FFB 07	FFP 04	FFP 04
P113	FFB 05	FFB 05	FFP 05	FFP 05
P114	FFB 06	FFB 06	FFP 04	FFP 04
P115	FFB 05	FFB 05	FFP 04	FFP 04
P116	FFB 05	FFB 05	FFP 05	FFP 05
P396	FFB 05	FFB 05	FFP 05	FFP 05
P457	FFB 05	FFB 05	FFP 04	FFP 04
P458	FFB 05	FFB 05	FFP 04	FFP 04
Favia gravida				
Abrolhos, Brazil				
B015	FFB 01	FFB 01	FFP 01	FFP 01
B016	FFB 01	FFB 01	FFP 01	FFP 01
B017	FFB 02	FFB 02	FFP 01	FFP 01
B018	FFB 01	FFB 01		
B042	FFB 01	FFB 01	FFP 01	FFP 01
B043	FFB 01	FFB 01	FFP 01	FFP 01
B072	FFB 01	FFB 01		
B073	FFB 01	FFB 01	FFP 02	FFP 02
B074	FFB 01	FFB 01		
B075	FFB 01	FFB 01	FFP 02	FFP 02
B079	FFB 01	FFB 01	FFP 02	FFP 02
B080	FFB 01	FFB 01	FFP 01	FFP 01
B081	FFB 01	FFB 01		
B082	FFB 01	FFB 01	FFP 01	FFP 01
B083	FFB 01	FFB 01		
B084	FFB 01	FFB 01		
B085	FFB 02	FFB 02	FFP 02	FFP 02
B086	FFB 01	FFB 01	FFP 01	FFP 01
B087	FFB 01	FFB 02	FFP 02	FFP 02

**Sample**	***β-tubulin***		***Pax-C***	
João Pessoa, Brazil				
B150	FFB 03	FFB 03	FFP 02	FFP 02
B162	FFB 04	FFB 04	FFP 02	FFP 02
B164	FFB 04	FFB 04	FFP 02	FFP 02
B170	FFB 04	FFB 04	FFP 02	FFP 02
B178	FFB 04	FFB 04	FFP 02	FFP 02
B209	FFB 01	FFB 01	FFP 01	FFP 01
B210	FFB 01	FFB 01	FFP 01	FFP 01
B211	FFB 01	FFB 01	FFP 01	FFP 01
B242	FFB 03	FFB 03		
B253	FFB 01	FFB 01	FFP 02	FFP 02
B254	FFB 01	FFB 01	FFP 02	FFP 02
B255	FFB 04	FFB 01	FFP 01	FFP 01
B256	FFB 03	FFB 03	FFP 02	FFP 02
B257	FFB 04	FFB 04	FFP 02	FFP 02
B258	FFB 01	FFB 01	FFP 02	FFP 02
B259	FFB 01	FFB 01	FFP 02	FFP 02
B261	FFB 01	FFB 01	FFP 01	FFP 01
B262	FFB 04	FFB 04	FFP 02	FFP 02
B263	FFB 04	FFB 04	FFP 02	FFP 02
B264	FFB 01	FFB 01	FFP 01	FFP 01
B265	FFB 01	FFB 01		
B266	FFB 04	FFB 04		
B300	FFB 04	FFB 04	FFP 02	FFP 02
B309	FFB 04	FFB 04	FFP 02	FFP 02
B313	FFB 04	FFB 04	FFP 02	FFP 02
B315	FFB 04	FFB 04	FFP 02	FFP 02
São Tomé, West Africa				
ST014	FFB 09	FFB 09	FFP 02	FFP 02
ST015	FFB 09	FFB 09	FFP 02	FFP 02
ST016	FFB 01	FFB 01	FFP 02	FFP 02
ST017	FFB 01	FFB 01	FFP 02	FFP 02
ST018	FFB 09	FFB 09	FFP 02	FFP 02
ST019	FFB 01	FFB 01	FFP 02	FFP 02
ST122	FFB 10	FFB 10	FFP 02	FFP 02
ST123	FFB 10	FFB 10	FFP 02	FFP 02
ST124	FFB 10	FFB 10	FFP 02	FFP 02
ST125	FFB 10	FFB 10	FFP 02	FFP 02
ST126	FFB 10	FFB 10	FFP 02	FFP 02
ST127	FFB 10	FFB 10	FFP 02	FFP 02
ST370	FFB 01	FFB 01	FFP 02	FFP 02
ST371	FFB 01	FFB 01	FFP 02	FFP 02
ST372	FFB 09	FFB 09	FFP 02	FFP 02

Sample	*β-tubulin*		*Pax-C*	
São Tomé, West Africa (cont.)				
ST373	FFB 09	FFB 01	FFP 02	FFP 02
ST374	FFB 01	FFB 01	FFP 02	FFP 02
ST375	FFB 01	FFB 01	FFP 02	FFP 02
ST376	FFB 01	FFB 01	FFP 02	FFP 02
ST377	FFB 01	FFB 01	FFP 02	FFP 02
Porites astreoides				
João Pessoa, Brazil				
B140	PAB 01	PAB 02		
B142	PAB 01	PAB 02		
B143	PAB 01	PAB 03		
B148	PAB 04	PAB 02		
B223	PAB 01	PAB 03		
B224	PAB 01	PAB 02		
B225	PAB 01	PAB 03		
B226	PAB 01	PAB 02		
B231	PAB 01	PAB 02		
B232	PAB 01	PAB 02		
B271	PAB 01	PAB 03		
B272	PAB 01	PAB 02		
B273	PAB 01	PAB 02		
B274	PAB 01	PAB 02		
B275	PAB 01	PAB 02		
B280	PAB 01	PAB 02		
B285	PAB 01	PAB 02		
B295	PAB 01	PAB 03		
B296	PAB 01	PAB 02		
B297	PAB 01	PAB 02		
B298	PAB 01	PAB 02		
Bocas del Toro, Panamá				
P004	PAB 05	PAB 02		
P018	PAB 02	PAB 06		
P058	PAB 05	PAB 02		
P059	PAB 02	PAB 06		
P136	PAB 05	PAB 06		
P137	PAB 05	PAB 07		
P138	PAB 05	PAB 07		
P139	PAB 02	PAB 06		
P140	PAB 05	PAB 02		
P141	PAB 05	PAB 02		
P142	PAB 05	PAB 05		
P143	PAB 02	PAB 06		

Sample	*β-tubulin*		*Pax-C*	
Siderastrea radians				
Abrolhos, Brazil				
B092	SB 201	SB 202	SP 201	SP 201
B093	SB 201	SB 202	SP 201	SP 201
João Pessoa, Brazil				
B133			SP 203	SP 203
B134			SP 203	SP 203
B146	SB 205	SB 205	SP 202	SP 202
B194	SB 205	SB 205	SP 201	SP 202
B283	SB 205	SB 205	SP 201	SP 201
Fortaleza, Brazil				
B358	SB 202	SB 208	SP 201	SP 204
B360	SB 202	SB 208	SP 201	SP 204
B361	SB 202	SB 208	SP 201	SP 204
B367	SB 202	SB 208	SP 201	SP 204
Bocas del Toro, Panamá				
P042	SB 202	SB 209	SP 206	SP 207
P043	SB 202	SB 209	SP 206	SP 207
P195	SB 210	SB 209	SP 201	SP 201
P196	SB 210	SB 211	SP 201	SP 201
P197	SB 202	SB 202	SP 201	SP 201
P198	SB 212	SB 213	SP 201	SP 201
P199	SB 210	SB 209	SP 201	SP 201
P200	SB 208	SB 214	SP 201	SP 208
P201	SB 202	SB 202	SP 201	SP 201
P202	SB 210	SB 209	SP 201	SP 201
São Tomé, West Africa				
ST010	SB 210	SB 202	SP 201	SP 202
ST024	SB 210	SB 202	SP 201	SP 204
ST034	SB 210	SB 202	SP 201	SP 202
ST075	SB 210	SB 202	SP 201	SP 202
ST076	SB 210	SB 202	SP 201	SP 202
ST077	SB 210	SB 202	SP 201	SP 202
ST078	SB 210	SB 202	SP 201	SP 202
ST133	SB 210	SB 202	SP 202	SP 202
ST223	SB 202	SB 206	SP 201	SP 202
ST224	SB 210	SB 202	SP 201	SP 202
ST225	SB 210	SB 202	SP 201	SP 202
ST226	SB 202	SB 206	SP 201	SP 202

Sample	*β-tubulin*		*Pax-C*	
São Tomé, West Africa (cont.)				
ST311	SB 203	SB 202	SP 201	SP 201
ST335	SB 210	SB 206	SP 201	SP 209
ST351	SB 202	SB 206	SP 201	SP 202
ST354	SB 210	SB 206	SP 201	SP 202
ST355	SB 202	SB 208	SP 201	SP 204
ST362	SB 210	SB 202	SP 201	SP 202
Siderastrea siderea				
Abrolhos, Brazil				
B096	SB 103	SB 104		
João Pessoa, Brazil				
B131	SB 105	SB 102	SP 101	SP 102
B135	SB 108	SB 109	SP 101	SP 101
B141	SB 111	SB 111	SP 101	SP 101
B149	SB 101	SB 114	SP 103	SP 103
B161	SB 103	SB 117	SP 103	SP 101
B185	SB 105	SB 105	SP 101	SP 101
B186	SB 118	SB 103	SP 103	SP 101
B192	SB 119	SB 119	SP 101	SP 102
B199	SB 104	SB 104	SP 101	SP 101
B240	SB 120	SB 108	SP 101	SP 102
B241	SB 108	SB 108	SP 101	SP 101
B245	SB 108	SB 108	SP 101	SP 102
B252	SB 108	SB 103	SP 101	SP 101
B277	SB 119	SB 119	SP 101	SP 102
Fortaleza, Brazil				
B325	SB 108	SB 108	SP 101	SP 102
B326	SB 108	SB 102	SP 101	SP 101
B327	SB 101	SB 108	SP 104	SP 105
B330	SB 122	SB 122	SP 102	SP 102
B332	SB 122	SB 119	SP 101	SP 101
B333	SB 108	SB 123	SP 101	SP 102
B345	SB 108	SB 123	SP 103	SP 101
B349			101	SP 102
B350	SB 108	SB 124	SP 101	SP 101
B355	SB 102	SB 102	SP 101	SP 101
Bocas del Toro, Panamá				
P007	SB 126	SB 127	SP 107	SP 103
P053	SB 128	SB 129	SP 108	SP 108
P054	SB 130	SB 131	SP 107	SP 101

Sample	*β-tubulin*		*Pax-C*	
Bocas del Toro, Panamá (cont.)				
P055	SB 132	SB 133	SP 106	SP 103
P088	SB 134	SB 135	SP 106	SP 103
P089	SB 103	SB 136	SP 103	SP 103
P090	SB 137	SB 138	SP 109	SP 109
P091	SB 139	SB 140	SP 107	SP 107
P093	SB 136	SB 143	SP 108	SP 108
P094	SB 144	SB 145	SP 103	SP 110
P095	SB 146	SB 147	SP 108	SP 101
P219	SB 148	SB 149		
P220	SB 150	SB 150		
P222	SB 151	SB 150		
P223	SB 150	SB 152	SP 111	SP 111
P224	SB 137	SB 153		
P502	SB 154	SB 155	SP 108	SP 108
P504	SB 156	SB 136		
São Tomé, West Africa				
ST011	SB 136	SB 136	SP 101	SP 101
ST012	SB 136	SB 136	SP 101	SP 101
ST025	SB 136	SB 136	SP 101	SP 101
ST031	SB 136	SB 136	SP 101	SP 101
ST079	SB 136	SB 136	SP 101	SP 101
ST119	SB 136	SB 136	SP 101	SP 101
ST120	SB 136	SB 136	SP 101	SP 101
ST121	SB 136	SB 136	SP 101	SP 101
ST134	SB 136	SB 136	SP 101	SP 101
ST137	SB 136	SB 136	SP 101	SP 101
ST144	SB 136	SB 136	SP 101	SP 101
ST145	SB 136	SB 136	SP 101	SP 101
ST190	SB 158	SB 136	SP 101	SP 101
ST191	SB 136	SB 136	SP 101	SP 101
ST198	SB 136	SB 136	SP 112	SP 112
ST292	SB 136	SB 136	SP 101	SP 101
ST314	SB 136	SB 136	SP 101	SP 101
ST349	SB 136	SB 160	SP 101	SP 101
***Siderastrea stellata* (*S. radians* x *S. siderea* hybrids)**				
Abrolhos, Brazil				
B094	SB 101	SB 101	SP 202	SP 202
B095	SB 102	SB 203	SP 201	SP 201
B097	SB 105	SB 106	SP 202	SP 202
B098	SB 105	SB 102	SP 202	SP 202
B099	SB 107	SB 204	SP 201	SP 201

Sample	β-tubulin		Pax-C	
João Pessoa, Brazil				
B136	SB 110	SB 205	SP 201	SP 203
B144	SB 205	SB 112	SP 203	SP 203
B145	SB 204	SB 113	SP 203	SP 203
B152	SB 115	SB 205	SP 203	SP 202
B156	SB 109	SB 205	SP 203	SP 203
B159	SB 116	SB 206	SP 201	SP 201
B198	SB 108	SB 207	SP 204	SP 204
B249	SB 121	SB 121	SP 205	SP 101
B284	SB 108	SB 207	SP 204	SP 204
Fortaleza, Brazil				
B359	SB 125	SB 206	SP 201	SP 202
B362	SB 125	SB 202	SP 201	SP 202
B363	SB 125	SB 202	SP 201	SP 202
B364	SB 125	SB 206	SP 201	SP 202
B365	SB 125	SB 206	SP 201	SP 202
B366	SB 125	SB 206	SP 201	SP 202
Bocas del Toro, Panamá				
P092	SB 141	SB 142	SP 106	SP 205
São Tomé, West Africa				
ST132	SB 157	SB 202	SP 201	SP 203
ST222	SB 158	SB 206	SP 201	SP 201
ST265	SB 136	SB 202	SP 201	SP 201
ST338	SB 159	SB 206	SP 201	SP 201
