# Supplementary material for: Long Distance Dispersal and Connectivity in Amphi-Atlantic Corals at Regional and Basin Scales
Source: PLoS One. 2011 Jul 22;6(7):e22298. doi: 10.1371/journal.pone.0022298 (PMC3142122; doi:10.1371/journal.pone.0022298)
Supplement: Table S4 — Genbank accession number for all sequenced haplotypes. (PDF) [file pone.0022298.s004.pdf]

F. gravida + F. fragum, β-tub

Haplotype	Accession Number
FFB01	HQ850473
FFB02	HQ850474
FFB03	HQ850475
FFB04	HQ850476
FFB05	HQ850477
FFB06	HQ850478
FFB07	HQ850479
FFB08	HQ850480
FFB09	HQ850481
FFB10	HQ850482

F. gravida + F. fragum, Pax-C

Haplotype	Accession Number
FFP01	HQ850452
FFP02	HQ850453
FFP03	HQ850454
FFP04	HQ850455
FFP05	HQ850456

Siderastrea radians, β-tub

Haplotype	Accession Number
SB201	HQ850495
SB202	HQ850496
SB203	HQ850497
SB204	HQ850498
SB205	HQ850499
SB206	HQ850500
SB207	HQ850501
SB208	HQ850502
SB209	HQ850503
SB210	HQ850504
SB211	HQ850505
SB212	HQ850506
SB213	HQ850507
SB214	HQ850508

Siderastrea radians, Pax-C

Haplotype	Accession Number
SP201	HQ850464
SP202	HQ850465
SP203	HQ850466
SP204	HQ850467
SP205	HQ850468
SP206	HQ850469
SP207	HQ850470
SP208	HQ850471
SP209	HQ850472

Porites astreoides - β-tub

Haplotype	Accession Number
PAB01	HQ850457
PAB02	HQ850458
PAB03	HQ850459
PAB04	HQ850460
PAB05	HQ850461
PAB06	HQ850462
PAB07	HQ850463

Siderastrea siderea, Pax-C

Haplotype	Accession Number
SP101	HQ850483
SP102	HQ850484
SP103	HQ850485
SP104	HQ850486
SP105	HQ850487
SP106	HQ850488
SP107	HQ850489
SP108	HQ850490
SP109	HQ850491
SP110	HQ850492
SP111	HQ850493
SP112	HQ850494

Siderastrea siderea, β-tub

Haplotype	Accession Number
SB101	HQ850509
SB102	HQ850510
SB103	HQ850511
SB104	HQ850512
SB105	HQ850513
SB106	HQ850514
SB107	HQ850515
SB108	HQ850516
SB109	HQ850517
SB110	HQ850518
SB111	HQ850519
SB112	HQ850520
SB113	HQ850521
SB114	HQ850522
SB115	HQ850523
SB116	HQ850524
SB117	HQ850525
SB118	HQ850526
SB119	HQ850527
SB120	HQ850528
SB121	HQ850529
SB122	HQ850530
SB123	HQ850531
SB124	HQ850532
SB125	HQ850533
SB126	HQ850534
SB127	HQ850535
SB128	HQ850536
SB129	HQ850537
SB130	HQ850538
SB131	HQ850539
SB132	HQ850540
SB133	HQ850541
SB134	HQ850542
SB135	HQ850543
SB136	HQ850544
SB137	HQ850545
SB138	HQ850546
SB139	HQ850547
SB140	HQ850548
SB141	HQ850549
SB142	HQ850550
SB143	HQ850551
SB144	HQ850552
SB145	HQ850553
SB146	HQ850554
SB147	HQ850555
SB148	HQ850556
SB149	HQ850557
SB150	HQ850558

Siderastrea siderea, β-tub (cont.)

Haplotype	Accession Number
SB151	HQ850559
SB152	HQ850560
SB153	HQ850561
SB154	HQ850562
SB155	HQ850563
SB156	HQ850564
SB157	HQ850565
SB158	HQ850566
SB159	HQ850567
SB160	HQ850568
